# Progressive pulmonary fibrosis due to diffuse alveolar damage in a COVID‐19‐infected autopsy case

**DOI:** 10.1002/rcr2.934

**Published:** 2022-03-16

**Authors:** Akiko Tamura, Ryosuke Imai, Yutaka Tomishima, Naoki Nishimura

**Affiliations:** ^1^ Department of Pulmonary Medicine St. Luke's International Hospital Tokyo Japan

**Keywords:** autopsy, COVID‐19, diffuse alveolar damage, pulmonary fibrosis

## Abstract

We encountered a patient with severe coronavirus disease 2019 (COVID‐19)‐related pneumonia, who died of progressive respiratory acidosis after 2 months of treatment with mechanical ventilation. The autopsy revealed diffuse alveolar damage (DAD) without any active signs of fungal or bacterial infections, suggesting prolonged and over‐activated immune responses against COVID‐19 infection. When COVID‐19 patients develop acute respiratory distress syndrome, it is essential to remember that the infection can progress to DAD a few months after the disease onset.

## Graphical abstract

We experienced a fatal case of coronavirus disease‐2019 (COVID‐19) pneumonia, who died of progressive respiratory acidosis after 2 months of treatment with mechanical ventilation. The autopsy revealed diffuse alveolar damage (DAD) without any active signs of fungal or bacterial infections, suggesting prolonged and over‐activated immune responses against COVID‐19 infection. When COVID‐19 patients develop acute respiratory distress syndrome, it is essential to remember that the infection can progress to DAD a few months after the disease onset.

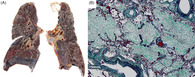

## INTRODUCTION

The coronavirus disease 2019 (COVID‐19) caused by the severe acute respiratory syndrome coronavirus 2 (SARS‐CoV‐2) has become a global pandemic, resulting in innumerable deaths. In severe cases of COVID‐19‐related pneumonia, acute respiratory distress syndrome (ARDS), histologically appearing as diffuse alveolar damage (DAD), is known to develop because of cytokine storms.^1^ Immunosuppressive agents are used for controlling cytokine storms, and the RECOVERY trial proved the prognostic benefit of steroid use.^2^ However, some patients show resistance to steroid therapy and require intensive care, including mechanical ventilation.^3^ According to previous autopsy case reports of COVID‐19 patients, most patients who presented with DAD died within 4 weeks; there are few reports of cases that presented with DAD and died more than 4 weeks after disease onset, and there are no detailed reports on the clinical course.^4^, ^5^

We report a fatal case of COVID‐19 with an autopsy finding in which the patient slowly developed progressive pulmonary fibrosis and respiratory acidosis after methylprednisolone therapy. The patient had died 58 days after the onset of COVID‐19 infection.

## CASE REPORT

A Japanese man in his 70s with a history of type II diabetes mellitus, hypertension and hyperlipidaemia, and a 15‐pack‐year smoking history, presented with a 1‐week history of cough and dyspnoea. He was diagnosed with COVID‐19 via a SARS‐CoV‐2 antigen test, which was verified by a polymerase chain reaction (PCR) test. He was intubated and admitted to our emergency room department for rapidly progressing respiratory failure.

On arrival, the patient was lethargic with a Glasgow Coma Scale score of E4VTM5, temperature of 37.2°C, blood pressure of 94/62 mmHg, pulse rate of 89 bpm, respiratory rate of 12/min and oxygen saturation of 88% on mechanical ventilation (fraction of inspired oxygen [FiO_2_]: 1.0). Coarse crackles were noted on physical examination. Laboratory findings revealed elevated a white blood cell count of 30,400/μl, haemoglobin level of 13.1 g/dl, platelet count of 110,000/μl, creatinine level of 1.36 mg/dl, haemoglobin A1c level of 9.0%, KL‐6 of 591 U/ml, C‐reactive protein level of 15.2 mg/dl, ferritin level of 2124 ng/ml and d‐dimer level of 22.0 μg/ml. Arterial blood gas testing showed a pH of 7.32, partial pressure of carbon dioxide (PaCO_2_) of 40.9 mmHg, partial pressure of oxygen (PaO_2_) of 62.0 mmHg, HCO^3−^ of 20.4 mEq/L and elevated lactate level of 2.1 mmol/L. Bilateral consolidation with lower lung predominance was observed on chest computed tomography (CT), consistent with the symptoms of COVID‐19 infection.

**FIGURE 1:**
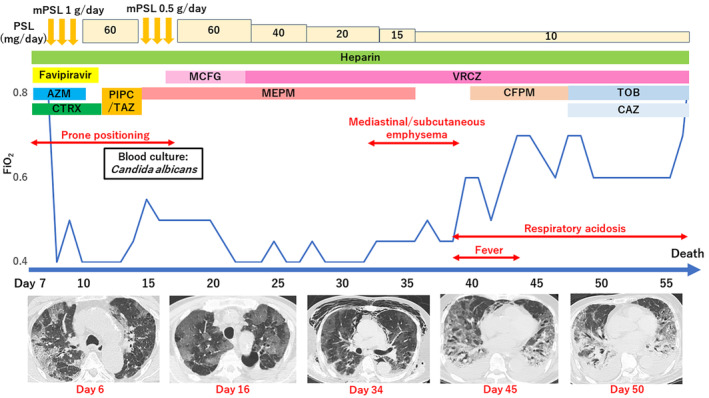
Clinical course. ‘Day X’ denotes the day since the patient's symptoms appeared. AZM, azithromycin; CAZ, ceftazidime; CFPM, cefepime; CTRX, ceftriaxone; FiO_2_, fraction of inspiratory oxygen; MCFG, micafungin; MEPM, meropenem; mPSL, methylprednisolone; PIPC/TAZ, piperacillin/tazobactam; PSL, prednisolone; TOB, tobramycin; VRCZ, voriconazole

He was admitted to the intensive care unit (ICU) and placed in a prone position. Continuous infusion of neuromuscular blocking agent and deep sedation were introduced. He was treated with pulsed methylprednisolone (1000 mg once daily) for 3 days and favipiravir (800 mg twice daily), in addition to the antibiotic therapy. Despite the decrease in his FiO_2_ requirement to 0.4, it worsened again on day 14 of symptom onset. His chest CT showed newly found diffuse bilateral ground‐glass opacities. Therefore, pulsed methylprednisolone was repeated from day 15 to day 17. At that time, his blood culture yielded positive results for *Candida albicans*. With the diagnosis of candidemia, he was initially administered empirical micafungin, which was later switched to voriconazole. After the initiation of antifungal therapy, his FiO_2_ level gradually improved. On day 33, he developed tachypnoea (respiratory rate, 35–40/min). His chest CT revealed mediastinal emphysema, which improved spontaneously by lowering positive end‐expiratory pressure (PEEP) and tapering the methylprednisolone to 10 mg/day. From day 39, he developed fever, and his FiO_2_ and PEEP requirement increased. Additionally, his tidal volume decreased to below 300 ml/breath, resulting in respiratory acidosis. Chest CT taken on day 44 demonstrated diffuse ground‐glass opacities along with bronchial dilatation and volume loss of bilateral lungs. On day 49, his blood pressure dropped, requiring vasopressor support, and his renal function deteriorated as respiratory acidosis progressed. The patient eventually died on day 58 (Figure 1).

**FIGURE 2:**
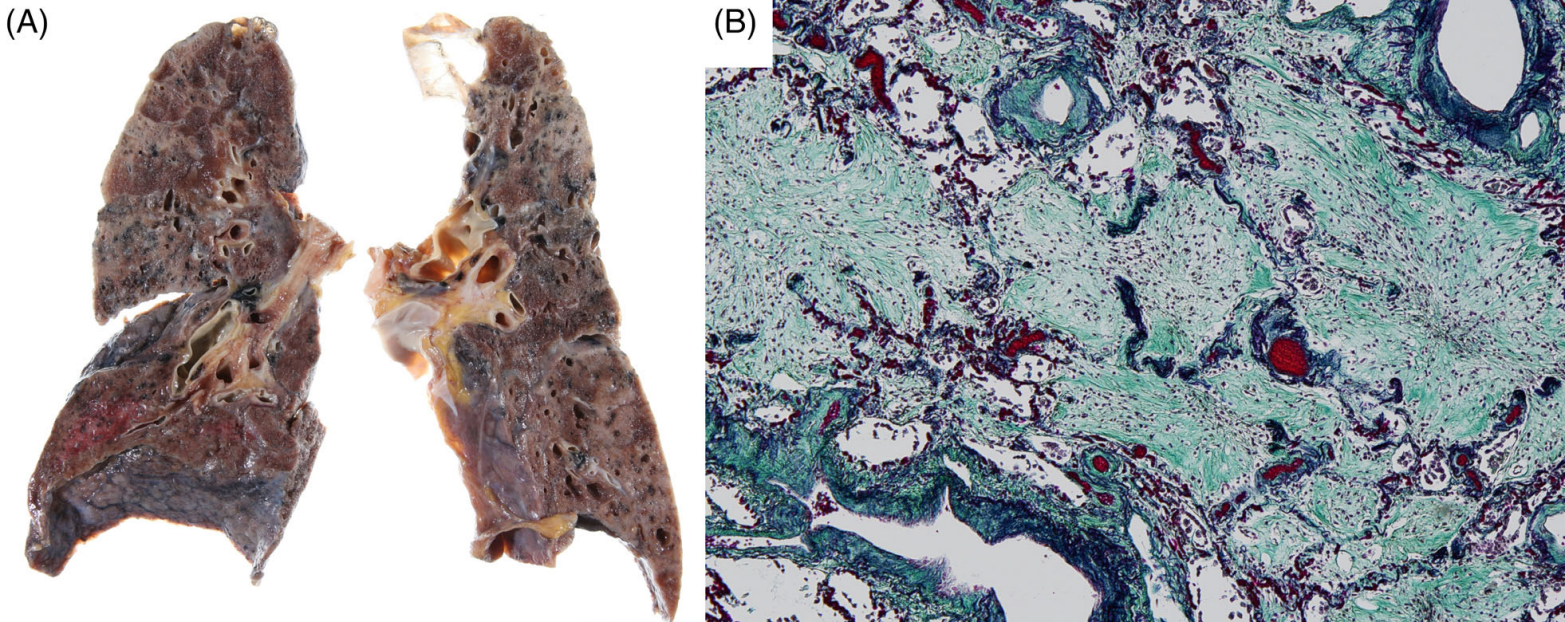
Gross appearance of both lungs. Both lungs had increased weight (right lung, 510 g; left lung, 450 g) (A). Microscopic appearance of the lung (Elastica‐Masson). Black alignment represents alveolar septum, filled with collagen fibre (B)

After his death, his family agreed to allow post‐mortem examination. The gross lung findings included increased lung weight (right lung: 510 g, left lung: 450 g) with solid appearance. Microscopic lung findings showed collapsed alveolar spaces filled with collagen fibres, consistent with the organizing phase of DAD. There was no evidence of active infection or embolism (Figure 2).

## DISCUSSION

In this report, the patient received pulsed methylprednisolone against ARDS due to COVID‐19. Although his respiratory condition temporarily improved, he eventually died of respiratory acidosis 58 days after the onset of COVID‐19, due to progression of pulmonary fibrosis.

It has been reported that in the ICU setting, approximately 60%–80% of COVID‐19 patients develop ARDS, with an average onset time of 8–9 days after the initial onset COVID‐19 symptoms. In previous autopsy reports, DAD has been the predominant histological finding, followed by organizing pneumonia, pulmonary embolism and alveolar haemorrhage.^1^ However, since most patients die within 1 month of onset and are autopsied, there are few reports on lung pathology persisting for more than 1 month after the disease onset.^4^, ^5^ In the study of autopsy cases of DAD with COVID‐19 by D'Agnillo et al., there was a marked deposition of viral antigen and activated neutrophils, along with increased levels of cytokines, chemokines, proteases, matrix metalloproteinases and cytotoxic reactive oxygen species found in alveolar and bronchiolar epithelial cells in corpses with the shortest interval from symptom onset to death. Meanwhile, corpses with a long interval from symptom onset to death showed a denuded respiratory epithelium and a lack of lung tissue repair and regeneration with prominent pulmonary fibrosis.^5^

In our patient's case, respiratory acidosis developed 40 days after the onset of symptoms, indicating that this restrictive change progressed over 1.5 months. In addition, SARS‐CoV‐2 PCR testing was continuously performed on tracheal aspirate during hospitalization, and the PCR continued to be positive for 30 days after disease onset, indicating that the SARS‐CoV‐2 viral load was persistently high, and this could have led to a sustained inflammatory response. We used corticosteroids and favipiravir as the initial treatment, followed by early reduction of corticosteroids. To improve the prognosis, we may have needed to use long‐term potent anti‐inflammatory treatment such as baricitinib (a JAK inhibitor) or tocilizumab (an IL‐6 inhibitor) as well as antiviral treatment such as remdesivir. Furthermore, in cases where fibrosis becomes a problem due to prolonged disease course, antifibrotic therapies are worth considering in addition to antiviral and anti‐inflammatory treatments. Clinical trials of antifibrotic therapies such as nintedanib and pirfenidone for progressing pulmonary fibrosis are currently underway, and these treatments may be an option in the future.^6^

Although candidemia occurred during the acute phase of ARDS, we consider that it only slightly contributed to the post‐acute pulmonary fibrosis, since antifungal therapy was initiated immediately with good response, and the progression of pulmonary fibrosis and respiratory acidosis was observed more than 1 month after its diagnosis. The recommended duration of therapy for candidemia without complications is 2 weeks. In this case, however, we had to continue administration of the antifungal drug. The reason for this was that the patient was still immunocompromised due to the continued use of corticosteroids for the treatment of pulmonary fibrosis, and his respiratory condition worsened around day 33; hence, we had to consider the possibility of a fungal breakthrough infection. As for bacterial infections, we empirically administered broad‐spectrum antibiotics when his respiration worsened, considering the risk of secondary bacterial infections. As it turned out, autopsy findings of the lungs did not show the presence of fungal organisms by Grocott's methenamine silver stain, and no neutrophilic infiltration or purulent exudates suggestive of fungal or bacterial infection were observed; therefore, the infection was considered to be well controlled. Furthermore, the involvement of ventilator‐induced lung injury should be considered in this case. Although preventative strategies were used such as limiting tidal volume inspiratory pressure, prone positioning and limit spontaneous respirator effort, lung injury may have progressed due to long‐term ventilator management. Certainly, we have not completely ruled out other infections or the effects of positive pressure ventilation. Based on the PCR results and lung autopsy findings, we think that SARS‐CoV‐2 itself was more involved than other infections in the progression of DAD.

In conclusion, we reported a lethal case of COVID‐19 with the prolonged progression of lung injury and respiratory acidosis over 2 months from symptom onset. It is important to keep in mind that DAD may develop over a long period of time after COVID‐19 infection.

## CONFLICT OF INTEREST

None declared.

## AUTHOR CONTRIBUTION

Akiko Tamura, Ryosuke Imai, Yutaka Tomishima and Naoki Nishimura managed the patient. Akiko Tamura and Ryosuke Imai drafted the manuscript, and Yutaka Tomishima and Naoki Nishimura edited and revised it critically.

## ETHICS STATEMENT

The authors declare that appropriate written informed consent was obtained for the publication of this manuscript and accompanying images.

## Data Availability

The data that support the findings of this study are available on request from the corresponding author. The data are not publicly available due to privacy or ethical restrictions.
